# Unpacking adiposity: a narrative critical appraisal of diverse obesity definitions

**DOI:** 10.1186/s13098-026-02261-6

**Published:** 2026-08-04

**Authors:** Marcio C. Mancini, Bruno Halpern, Maria Edna de Melo, Caroline K. Kramer

**Affiliations:** 1 Department of Endocrinology, Universidade de Sao Paulo, Sao Paulo, Brazil; 2 Associação Brasileira para o Estudo da Obesidade e Síndrome Metabólica, Sao Paulo, Brazil; 3 University of Toronto, Sinai Centre for Diabetes at Mount Sinai Hospital, Toronto, Canada

**Keywords:** Adipose tissue dysfunction, Body mass index, Clinical obesity, Controlled obesity, Diet quality, Immunometabolism, Metabolic syndrome, Reduced obesity, Type 2 diabetes, Waist circumference

## Abstract

**Background:**

Obesity is a complex, multifaceted chronic disease that poses a major global health challenge. Traditionally, population-level screening and follow-up have relied heavily on body mass index (BMI). Although BMI provides a simple, standardized measure for epidemiological studies, it does not account for individual variations in muscle mass, fat distribution, or metabolic health. Consequently, relying solely on weight-to-height ratios—even when supplemented with waist circumference, waist-to-hip ratio, or waist-to-height ratio—limits precise individual diagnosis and comprehensive health risk assessment. Comprehensive risk stratification incorporating clinical factors beyond body weight is therefore needed to capture the heterogeneity of obesity and enable a more individualized, patient-centered approach to management.

**Main body:**

This critical narrative review evaluates the main definitions and classification systems used for obesity, including the traditional World Health Organization classification, the Edmonton Obesity Staging System (EOSS), the Lancet Commission framework, the European Association for the Study of Obesity framework, and classifications based on weight history. BMI remains valuable for population-level assessment and may also be useful at the individual level when combined with additional anthropometric measurements and sex-, age-, and ethnicity-specific cutoff values. The EOSS complements anthropometric assessment by incorporating metabolic, physical, psychological, and functional consequences, thereby supporting treatment intensity according to disease severity. The Lancet Commission definitions of preclinical and clinical obesity broaden diagnosis beyond BMI but have raised concerns regarding the absence of standardized screening tools, potential diagnostic inconsistency, and possible delays in treatment for people classified as having preclinical obesity. Such limitations may be particularly relevant for children and adolescents, in whom delayed intervention may increase long-term health risks. Weight-history classifications provide an additional perspective by incorporating maximum weight attained in life, supporting realistic treatment goals, reinforcing the chronic nature of obesity, reducing stigma, and encouraging long-term treatment adherence.

**Conclusion:**

No single classification adequately captures the biological and clinical heterogeneity of obesity. BMI should not be abandoned, but interpreted alongside anthropometric, metabolic, functional, psychological, and weight-history information. Integrating complementary classification systems may improve diagnostic accuracy, risk stratification, treatment prioritization, and patient-centered obesity care.

## Background

Obesity is largely diagnosed based on a simple clinical measure, body mass index (BMI) [1, 2]. However, excess body weight alone does not necessarily translate to adverse health outcomes, and having a normal BMI does not always mean good metabolic health. Individuals with the same BMI can fall into different disease risk categories, as factors beyond body weight contribute to disease risk [3–6] and additional measures can be useful. Therefore, a one-size-fits-all approach is not sufficient, and a more individualized and targeted approach is needed to identify those at higher risk for adverse outcomes and ensure they receive the most appropriate obesity treatment.

To ensure a comprehensive and balanced appraisal, a nonsystematic literature search was conducted in the PubMed/MEDLINE, Embase, and Google Scholar databases for articles published up to May 2026. Search terms included combinations of ‘obesity definition’, ‘obesity classification’, ‘adiposity staging’, ‘body mass index limitations’, and ‘metabolic health’. Frameworks were selected for inclusion based on three objective criteria: [1] formal endorsement or development by major international medical societies (WHO, EASO); [2] prominent citation and clinical validation in the literature (Edmonton Obesity Staging System, Lancet Commission); or [3] a weight history approach. By examining these specific systems, this critical appraisal aims to contrast globally recognized benchmarks with emerging clinical models to map the evolution of obesity stratification.

## World Health Organization definition

Obesity is defined by the World Health Organization as “abnormal or excessive fat accumulation that presents a risk to health”. Although it is a simple definition, what constitutes “abnormal or excessive” fat or what is “risk to health” can be broadly interpreted.

Putting this into historical context, almost 30 years ago, the World Health Organization (WHO) Consultation on Obesity assembled a group of more than 100 experts worldwide, chaired by Philip James, to review epidemiological information on obesity and make recommendations for the development of public health policies and programs for the prevention and management of obesity. To develop comprehensive public health policies and strategies for the prevention and management of obesity, they evaluated factors contributing to obesity and its associated consequences, including chronic noncommunicable diseases and their health and economic effects. The report recognizes obesity as a serious, chronic, complex, and not fully understood disease [1].

The key issues covered include the graded classification of overweight and obesity, enabling meaningful comparisons of weight categories within and between populations, identifying individuals and groups at increased risk of morbidity and mortality, and providing a firm basis for the evaluation of interventions [1].

### Body mass index

The mathematical formula *weight/height*^*2*^ was originally developed by the Belgian mathematician and sociologist Lambert Adolphe Jacques Quételet in his work *Sur l’homme et le development de ses facultés* published in 1832. Quételet had a passionate interest in physical characteristics and social aptitudes, seeking the proportion of what would be an "average man" (*l’homme moyen*) [7]. The modern term (BMI) was coined in a paper published in 1972 by Ancel Keys and others, arguing that what they termed BMI was "if not fully satisfactory, at least as good as any other relative weight index as an indicator of relative obesity", and explicitly judging BMI appropriate for population studies while inappropriate for individual clinical assessment [8, 9].

BMI is a simple index of weight-for-height that is commonly used to classify underweight, overweight, and obesity in adults, defined as the weight in kilograms divided by the square of the height in meters (kg/m^2^). Due to its simplicity, it has become widely used for preliminary diagnosis [10], remaining a useful initial tool for comprehensive risk assessment [11].

The WHO report considers BMI a very useful, albeit crude, population-level measure of obesity, used to estimate the prevalence of obesity and the risks associated with it. The report recognizes, however, that BMI does not account for the wide variation in body fat distribution [5, 6, 9, 12]. In this context, measurement of waist circumference (WC) provides a simple and practical method of identifying overweight patients at increased risk of obesity-associated illness due to abdominal fat distribution. It is also important to consider that ethnic populations differ in the level of risk associated with a given WC [4–6, 9, 12]. Other tools are available for a more detailed characterization of these fat depots, including methods that measure body composition (e.g., underwater weighing), and locate the anatomical distribution of body fat (e.g., magnetic resonance imaging or computed tomography), but the cost of such techniques limits their use in clinical practice [1].

Longitudinal studies confirm the utility of BMI for assessing long-term health outcomes, but its accuracy can be limited by variations across age and ethnicity, with some groups having different health risks at similar BMI levels. While epidemiological data show a population-level U-shaped relationship between BMI and risk (lowest risk in a mid-range), it is best used as a screening tool rather than as a standalone diagnostic tool, as it does not account for body composition or distribution of fat [13–22]. Table 1 presents a comparison of longitudinal studies indicating the association of BMI with long-term health outcomes.

**Table 1:** Longitudinal studies evaluating the association between BMI and long-term health outcomes

Reference	Main outcome measure	Study cohort/population	Main results and conclusions
[13]	Relationship between BMI and all cause mortality adjusted for age, study, physical activity, alcohol consumption, educational and marital status	Pooled data from 19 prospective studies including 1.46 million White adults aged 19–84 years (median age, 58 years), median BMI 26.2 kg/m^2^, follow-up 10 years (range 5–28)	Lowest all cause mortality was observed at a BMI of 20.0 to 24.9 kg/m^2^
[14]	Associations between BMI and all-cause and cause-specific mortality adjusted for age, sex, smoking status and study	Collaboration of 57 prospective studies with 894,576 participants primarily from western Europe and North America (61% male, mean age 46 years, mean BMI 25 kg/m^2^)	Lowest mortality was observed at a BMI of 22.5–25 kg/m^2^
[15]	Influence of BMI on the risk of death from cancer	Prospective study population of 900,053 US adults free of cancer at enrollment in 1982 with 57,145 cancer deaths during 16 years of follow-up	Relative risk of cancer-related death increased by 52% in men and 62% in women (esophagus, colon and rectum, liver, gallbladder, pancreas, kidney, non-Hodgkin’s lymphoma, and multiple myeloma)
[16]	Influence of BMI on the risk of death from any cause	Prospective study of 527,265 US adults NIH-AARP cohort, aged 50–71 years at enrollment (1995–1996) with 10 years of follow-up and 61,317 deaths	The relative risk of death increased by 96% in people with class I obesity, 146% in class II obesity, and 282% in class III obesity
[17]	Meta-analyses with adjustment for study, age and sex	189 prospective studies from Asia, Australia, Europe and North America of BMI with 951,455 never-smokers without chronic diseases at recruitment (median follow-up 13.7 years)	All-cause mortality was lowest at a BMI of 20.0–25.0 kg/m^2^ and increased by 13–51% below or and above this range, by 7–20% among individuals with overweight, by 45% among class I obesity, by 94% among class II, and by 176% among class III
[18]	Associations between BMI and all-cause and specific-cause mortality	UK primary care data from CPRD and national mortality registration data recorded by ICD-10, follow-up from 1998 to 2016, adults aged 40 years at baseline; 3,632,674 people included, being 1,969,648 never-smokers, with 188,057 deaths	Hazard ratio for death including deaths from mental, behavioral, neurological and external causes, increased by 21% per 5 kg/m^2^ above 25 kg/m^2^. Life expectancy from age 40 years was 4.2 years shorter in women and 3.5 years shorter in men with obesity
[19]	Association of BMI in midlife with morbidity, longevity and health care expenditure	Prospective cohort study of 29,621 adults (57.1% men) with a mean baseline age of 40 years who were at least 65 years old at follow-up, Chicago Heart Association Detection Project Industry with in-person examination (1967–1973) and Medicare follow-up (1985–2015)	Higher cumulative morbidity burden in older adulthood among overweight (7.2 morbidity-years) and classes I/II obesity (9.8) compared with normal BMI (6.1) in midlife. Age at death was similar between overweight (82.1 years old) and normal BMI (82.3), but shorter in those with class I/II (80.8). Cumulative excess health costs after 65 years were higher for individuals who were overweight or had obesity in midlife
[20]	Estimation of years of life lost due to obesity across the life span	Data from the US Life Tables (1999), NHANES III (1988–1994), NHANES I and II (1971–1992) n = 14,407, and NHANES II Mortality Study (1976–1992), n = 9,252	The BMI associated with the greatest longevity was approximately 23–25 for White individuals and 23–30 for Black individualss. For any degree of overweight, younger adults had greater years of life lost due to obesity than did older adults
[21]	To assess the relative risk of mortality by BMI in whites and blacks among subgroups defined by smoking, prevalent disease and age	Cancer Prevention Study enrolled 929,691 participants in 1982, with 28 years of follow-up with 453,102 deaths	Optimal health and longevity was observed among individuals with a BMI between 20.0 and 25.0 kg/m^2^
[22]	Systematic review and meta-analysis of cohort studies of BMI and risk of all-cause mortality	230 cohort studies (207 publications), including 53 studies of never-smokers with 9,976,077 participants and 738,144 deaths	Relative risk associated with a 5-unit increase in BMI was 18% among never-smokers, 21% among healthy never-smokers, 27% among healthy never-smokers excluding early follow-up. Lowest risk observed at BMI 22–23 kg/m^2^

CPRD: Clinical Practice Research Datalink; ICD-10: International Classification of Diseases, 10th revision codes; NHANES II: First National Health and Nutrition Examination Survey; NHANES II Mortality Study; NHANES III: Third National Health and Nutrition Examination Survey; NIH-AARP: National Institute of Health-American Association of Retired Persons

### Other anthropometric indexes

The WHO classification of overweight and obesity according to BMI is primarily based on the association between BMI and mortality and is shown in Table 2. The risks associated with increasing BMI are continuous and graded and begin at a BMI greater than 25 [13–22]. The interpretation of BMI categories in relation to risk may differ among populations [17]. Both BMI and a measure of fat distribution (WC or waist-hip ratio [WHR]) are important in assessing the risk of obesity-related comorbidities [23–27]. A high WHR (WHR  > 1.0 in men and  > 0.85 in women) indicates fat accumulation [1, 27], but WC alone—measured at the midpoint between the lower border of the rib cage and the iliac crest—provides a useful, inexpensive, and simple correlate of abdominal fat distribution and the associated risk of adverse health outcomes [23–27]. In 1996, Ashwell et al. proposed the waist-to-height ratio (WHtR) as a better anthropometric predictor of intra-abdominal fat and a stronger predictor of cardiometabolic disease risk in adults. Furthermore, the ratio of WC to height is similar between the sexes, thus eliminating the need for sex-specific cutoff points [28]. WHtR has been shown to be a better surrogate measure of adiposity than other anthropometric indices in adults [29], children, and adolescents [30], as well as a better predictor of visceral adipose tissue than WC, WHR, and BMI [30, 31], with a universal cutoff point of 0.5 having been suggested [27, 32].

**Table 2:** Classification of adults according to the body mass index (BMI)

Classification	BMI (kg/m^2^)	Risk of obesity-related comorbidities
Underweight	< 18.5	Low
Normal weight	18.5- < 25	Average
Excess of weight	≥ 25	
Overweight	25- < 30	Increased
Obesity class I	30- < 35	Moderate
Obesity class II	35- < 40	Severe
Obesity class III	≥ 40	Very severe

An important consideration pertains to ethnicity. The WHO document states that people of South Asian descent living in urban societies have a higher prevalence of many obesity-related complications than other ethnic groups. In addition, this ethnic group usually presents with a markedly greater amount of abdominal fat for a given BMI than Europeans [1, 33, 34]. This is particularly important because the original BMI cutoff values were derived from studies in Caucasian populations. Recently, a large study conducted in England, demonstrated that Caucasians, compared with other ethnic groups, are the outliers. Specifically, individuals of Black, Chinese, Arab, and South-Asian ancestry had an increased diabetes risk at much lower BMI thresholds. Revisions of ethnicity-specific BMI cutoffs are needed to ensure that minority ethnic populations are provided with appropriate screening and prevention of obesity-related complications [35]. Well-known studies from the Japanese American Community Diabetes Study demonstrated that excess visceral adiposity is a key determinant of increased metabolic risk among second- and third-generation Japanese Americans, independent of overall obesity [36, 37].

With regard to sex differences, the sex-specific WC thresholds associated with a higher risk of metabolic complications in Whites were established in a study of a randomly selected convenience sample from the civil registry of Amsterdam and Maastricht (Netherlands) of 2,183 men and 2,698 women aged 20–59 years. Despite the characteristics of the study population, these cutoff limits are still adopted by most commonly used definitions of metabolic syndrome for people of European ancestry [38, 39]. According to this study, the risk of metabolic complications iincreases when WC is greater than or equal to 94 cm in men and 80 cm in women, and increases further when it is greater than or equal to 102 cm and 88 cm, respectively [38, 39].

The INTERHEART study was a standardized case–control study of acute myocardial infarction with more than 27,000 participants in 52 countries representing various major ethnic groups and assessed the relationships among BMI, WC, hip circumferences, WHR, and myocardial infarction. Interestingly, when BMI quintiles were evaluated, there was a modest and graded association with myocardial infarction that became nonsignificant after adjustment for eight other risk factors, namely, apolipoproteins A and B, history of hypertension, history of diabetes, diet, activity, alcohol use and psychosocial variables. In contrast, for WHR showed a stepwise association across quintiles, even after adjustment for other risk factors. The population-attributable risk of myocardial infarction associated with an increased WHR in the top two quintiles was more than 24% compared with less than 8% for the top two quintiles of BMI. The WHR was more strongly and consistently associated with myocardial infarction risk than BMI across sex, age, and risk-factor subgroups, likely because it better captures body fat distribution by accounting for both abdominal and pelvic girth; notably, larger hip circumference showed an inverse association with myocardial infarction risk after BMI adjustment [40].

ELSA-Brasil is a large, ongoing prospective cohort study designed to investigate the determinants and consequences of chronic diseases, particularly cardiovascular disease and diabetes, among Brazilian adults [41]. One study showed that greater lower-body relative to trunk fat is associated with lower 10-year cardiovascular risk and a more favorable risk-factor profile, particularly among women [42]. One longitudinal analysis provided robust evidence that WHtR outperforms BMI and WC in predicting incident coronary artery calcium, particularly among individuals with a BMI below 30 kg/m^2^ [43].

In accordance with the International Diabetes Federation definition of metabolic syndrome, WC values ​​are ethnicity-specific. For populations of European ancestry, the established cutoffs for men and women are  ≥ 94 cm and  ≥ 80 cm, respectively. In contrast, in the United States, the National Cholesterol Education Program Adult Treatment Panel III cutoff values of  > 102 cm for men and  > 88 cm for women remain in use, with the caveat that some male patients may develop metabolic risk factors even at marginally increased values between 94 and 102 cm. Thresholds of  ≥ 94 cm for men and  ≥ 80 cm for women apply to Sub-Saharan African, Eastern Mediterranean, and Middle Eastern (Arab) populations. Conversely, cutoffs of  ≥ 90 cm for men and  ≥ 80 cm for women are used for South Asian, Chinese, Malay, Asian-Indian, Japanese, and ethnic South and Central American populations until further data become available [39].

Obesity-driven complications are fundamentally rooted in adipose tissue dysfunction (adiposopathy) rather than absolute body mass. Traditional metrics such as BMI fail to capture the cellular and immunometabolic state of adipose tissue. When positive energy balance induces pathological adipose tissue expansion, adipocyte hypertrophy outpaces angiogenesis, causing local hypoxia, cell death, and altered adipokine secretion (such as elevated leptin and suppressed adiponectin). This microenvironment triggers a chronic, low-grade inflammatory cascade characterized by massive macrophage infiltration and insulin resistance, accelerating the development of type 2 diabetes and metabolic syndrome [44]. This concept acknowledges that two individuals with identical BMIs may possess completely distinct immunometabolic profiles, thereby requiring fundamentally different clinical monitoring and management strategies [45, 46].

Individuals categorized into identical BMI classes can also present vastly different metabolic risks depending on their consumption of ultra-processed foods versus cardiometabolic or Mediterranean-style dietary patterns. Recent longitudinal data highlight that greater adherence to a Western dietary pattern strongly predicts a higher risk of metabolic syndrome and insulin resistance, whereas a prudent, nutrient-dense diet serves as a protective factor [47]. This emphasizes that changing the focus from simple calorie restriction to high-quality dietary adjustments is essential for mitigating cardiometabolic risks. Furthermore, the success of weight loss strategies depends closely on macronutrient quality. Clinical evidence reveals that low-carbohydrate diets emphasizing healthy, plant-based proteins and fats are associated with significantly slower long-term weight gain compared with animal-based low-carbohydrate dietary patterns [48]. Similarly, specialized strategies such as the high-polyphenol ‘Green-Mediterranean’ diet have shown unique efficacy in targeting visceral adiposity and shifting gut microbiome profiles toward improved metabolic pathways [49].

The WHO document identifies the establishment and validation of BMI standards for older adults as priority areas for further research [1]. With aging, changes in body composition occur, especially loss of lean body mass and bone mass, and an increase in fat mass, particularly visceral fat mass. Changes in body composition among older adults are documented primarily by cross-sectional studies [50–52], but also by longitudinal studies [53–55]. In addition to the metabolic consequences of obesity, in the elderly individual, excess weight overloads the musculoskeletal system, being a major risk factor for osteoarthritis, thus impairing physical functioning and leading to mobility limitations, which are common among older people with obesity. On the other hand, it is argued that there could be some protective effect of a certain amount of excess weight, including greater nutritional reserves, greater hemodynamic stability, increased bone mineral density, and protection provided by fat around the hip area in the event of a fall. Although there is no precise threshold, for adults aged 65 years and older, the normal BMI range is slightly higher than what is considered normal for younger adults [56]. According to a meta-analysis that included 32 studies with 197,940 individuals with an average follow-up of 12 years, evaluating the association between BMI and all-cause mortality in adults  ≥ 65 years of age, an ideal BMI for older adults may be slightly higher, potentially 23–27 kg/m^2^. A BMI below 23 and above 33 kg/m^2^ is linked to an increased risk of mortality [57]. In older adults, however, caution regarding reverse causation is warranted, as short-term studies can capture higher risks at lower BMIs due to unintentional weight loss related to ongoing illness [58]. In this vulnerable population, standard BMI parameters entirely fail to capture the clinical reality of low muscle mass coupled with high adiposity. Consequently, nutritional staging must focus explicitly on protein adequacy and targeted resistance exercise. The primary nutritional goal in these patients must shift from aggressive energy restriction to preserving lean mass and physical function during weight reduction, preventing the exacerbation of sarcopenia [56].

## Edmonton obesity staging system

It is well established that excess body fat commonly results in impaired health, reduced quality of life and well-being, and increased morbidity and mortality. Although increased body fat can have important implications for health, the mere presence of increased body fat does not necessarily imply or reliably predict disease. Thus, the current anthropometric classification systems, based on simple clinical measures, such as height, weight, or WC, do not necessarily reflect the presence or severity of obesity-related health risks, comorbidities, or reduced quality of life. Accurate risk prediction requires distinguishing adiposity from lean mass, which BMI cannot achieve. As a result, individuals with high BMI but low fat mass and those with normal BMI but excess adiposity may be misclassified [2–4, 59].

The proposal for a clinical and functional staging system for obesity is analogous to classification systems used for other diseases, providing a framework in which health professionals can document and report the extent and severity of disease. The TNM (tumor, node, metastasis) classification is widely used in oncology to designate the degree of tumor expansion and spread in most types of cancer. The New York Heart Association (NYHA) functional classification system is widely used to classify the severity of heart failure, helping to determine the best course of therapy. It reports symptoms and their impact on daily activities and quality of life in patients with congestive heart failure, ranging from class I, (mild) to class IV (severe). The Kidney Disease Outcomes Quality Initiative (KDOQI) classification categorizes renal function from stage 1 (normal function) to stage 5 (end-stage renal disease) based on the glomerular filtration rate [60].

Staging systems permit appropriate stratification of disease severity and its consequences, enabling the classification of patients according to risk. Additionally, they facilitate communication among health professionals, thereby facilitating the implementation of clinical guidelines and treatment allocation. In contrast, the anthropometric definition of obesity provides little guidance regarding individual risk, treatment strategies or the assessment of treatment outcomes [60].

The rationale for a clinical staging system is based on the premise that patients with obesity-related health complications should be treated more aggressively. Furthermore, in situations of limited financial resources, a staging system can help identify patients who are most likely to benefit from weight management [59, 60].

The Edmonton Obesity Staging System (EOSS), proposed in 2009, is based on simple clinical evaluations, including medical history, clinical and functional assessment, and readily available routine diagnostic examinations. It indicates the presence and severity of risk factors, obesity-associated diseases and functional limitations, thereby serving as a guide to patient management. The EOSS is a five-stage obesity classification system that considers metabolic, mechanical, and mental parameters to determine the most appropriate obesity treatment (Table 3) [59].

**Table 3:** Edmonton Obesity Staging System (EOSS)

EOSS stage	Description	Management
0	No apparent obesity-related risk factors, physical symptoms, psychopathology, functional limitations, and/or impairment of well-being	Identification of factors contributing to increased body weight. Counseling to prevent further weight gain through lifestyle measures, including healthy eating and increased physical activity
1	Presence of obesity-related subclinical risk factors, mild physical symptoms, mild psychopathology, mild functional limitations, and/or impairment of well-being	Investigation of other (non-weight related) contributors to risk factors. More intense lifestyle interventions, including diet and exercise, to prevent further weight gain. Monitoring of risk factors and health status
2	Presence of obesity-related chronic disease, moderate limitations in activities of daily living, and/or impairment of well-being	Initiation of obesity treatment*. Close monitoring and management of comorbidities as indicated
3	Established end-organ damage, significant psychopathology, significant functional limitations, and/or impairment of well-being	More intensive obesity treatment*. Aggressive management of comorbidities as indicated
4	Severe (potentially end-stage) disabilities resulting from obesity-related chronic diseases, disabling psychopathology, functional limitations, and/or impairment of well-being	Aggressive obesity management as deemed feasible. Palliative measures, including pain management, occupational therapy, and psychosocial support

^*^ Including consideration of all behavioral, pharmacological, and surgical treatment options

**Fig. 1:**
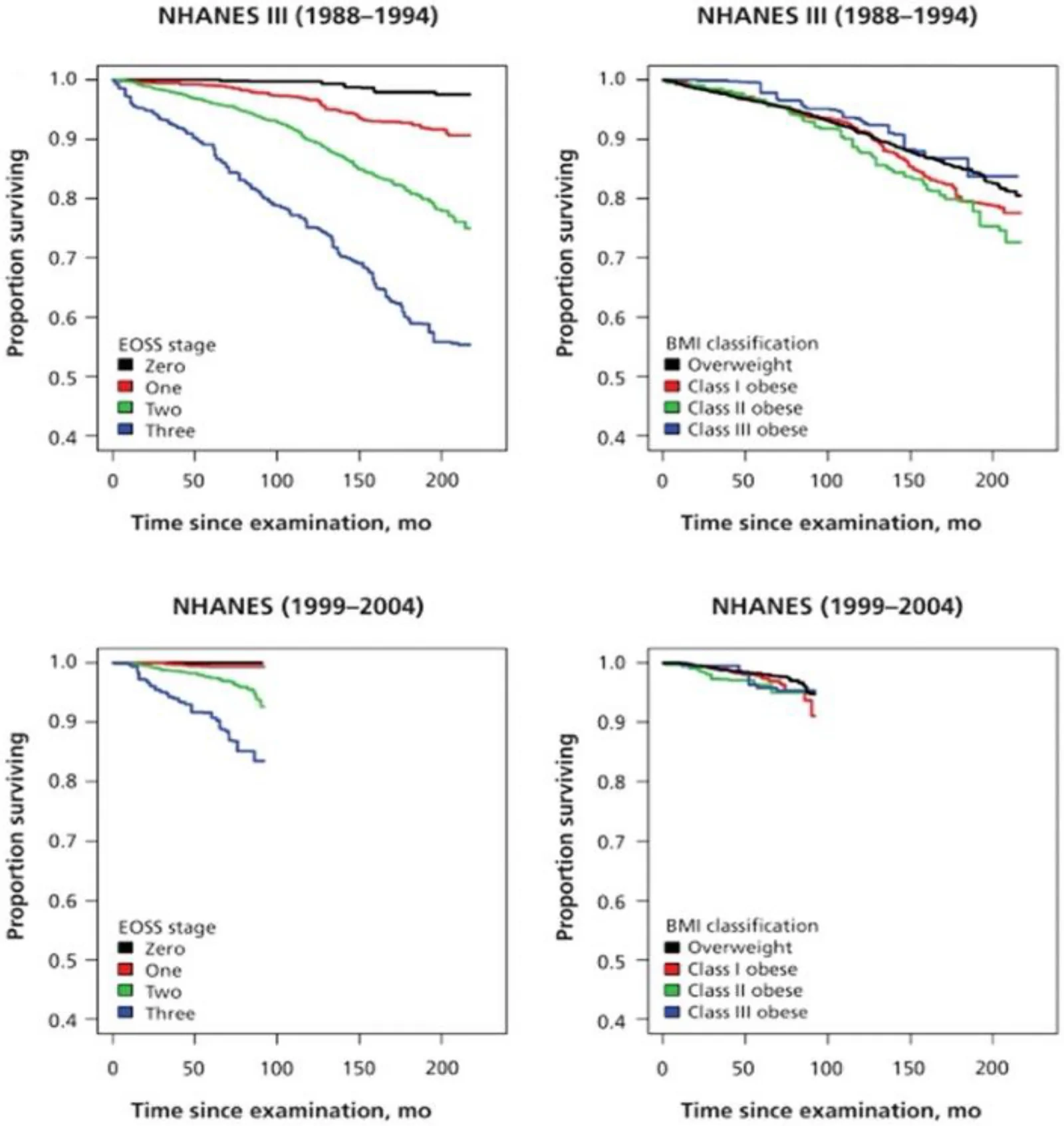
Kaplan–Meier plots examining all-cause mortality according to Edmonton obesity staging system and anthropometric classification among people with overweight and obesity. BMI = body mass index, EOSS = Edmonton obesity staging system, NHANES = National Health Nutrition Examination Surveys. Source [60]:

The EOSS was able to predict mortality in a nationally representative US sample of adults with overweight or obesity (data from the National Health and Nutrition Examination Surveys III 1988–1994 and 1999–2004), with mortality follow-up through the end of 2006. Over 75% of the cohort had scores of 1 or 2. Score 4 could not be reliably assigned because specific data elements were lacking. Survival curves clearly diverged when stratified by EOSS scores of 0–3, but not when stratified by obesity class alone (Fig. 1), confirming that the EOSS offers improved clinical utility in assessing obesity-related risk and prioritizing treatment [60]. Likewise, higher EOSS scores are associated with perioperative complications following bariatric and metabolic surgery [61, 62].

The EOSS does have some potential disadvantages. This is not a dynamic classification system; although some complications may improve with treatment, leading to a theoretical reduction in the EOSS score, many of the criteria are static and nonmodifiable. Therefore, it is not a useful tool for assessing long-term outcomes. It is also important to discuss whether the complications that lead to a higher EOSS stage are directly caused by obesity and whether treating obesity can improve these complications, such as diabetic retinopathy (a criterion for EOSS stage 3).

## Definition and diagnostic criteria of clinical obesity

In 2023, a Commission on Clinical Obesity was established through collaboration between *The Lancet Diabetes & Endocrinology* and the Institute of Diabetes, Endocrinology and Obesity at King’s Health Partners. The Commission comprised nearly 60 experts and was chaired by Francesco Rubino, Professor and Chair of Metabolic and Bariatric Surgery at King’s College London [63].

The final document [64] was published in 2025 with the endorsement of at least 70 medical societies, including The Obesity Society, American Association of Clinical Endocrinologists, the American Diabetes Association, World Obesity Federation, and Brazilian Association for the Study of Obesity and Metabolic Syndrome (ABESO). In contrast, the Obesity Medicine Association [65] and the European Association for the Study of Obesity [66] issued statements expressing opposition to the report.

It is important to acknowledge that endorsement of the document by a society or federation does not imply unconditional agreement or suggest that this proposal should immediately replace any other diagnostic methods. It should be recognized, however, that that the process in which the document was developed was of high scientific quality and that the document contains several strengths that deserve broad recognition, including the importance of moving beyond BMI alone as the sole diagnostic tool for obesity.

### Diagnosing obesity, clinical obesity and preclinical obesity

The Commission presented a transformative framework defining obesity as a spectrum of disease states, introducing the concepts of preclinical obesity and clinical obesity, recognizing the limitations of BMI in correctly identifying individuals with excessive adipose tissue, and proposing a functional approach that assesses organ and tissue dysfunction [63].

The Commission proposed updated conceptual definitions, emphasizing that obesity is defined by excess adiposity, regardless of whether adipose tissue distribution or function is altered. It distinguished clinical obesity as a chronic, systemic disease in which excess adiposity disrupts the function of organs and tissues, leading to recognizable clinical manifestations or functional limitations, whereas preclinical obesity refers to excess adiposity without evident organ dysfunction. Pre-obesity was described as an earlier point along the continuum of increasing body adiposity. These concepts were subsequently operationalized through specific diagnostic criteria [64].

The report recommends adopting one of the following three criteria for the diagnosis of obesity: direct body fat measurement (by dual-energy X-ray absorptiometry or bioimpedance analysis), or one anthropometric criterion (WC, WHR, or WHtR) in conjunction with BMI, or two anthropometric criteria without considering BMI (age-, gender-, and ethnicity-appropriate cutoff points should be used for all criteria). Accordingly, obesity can no longer be diagnosed solely on the basis of a high BMI; a professional diagnosis will now require additional assessments that confirm excess body fat and its associated health risks [64].

Although the limitations of using BMI alone are well recognized and identified, the fact that three distinct criteria coexist for diagnosing obesity in a single individual may result in situations in which an individual may or may not receive a diagnosis of obesity, depending on the criteria adopted. Furthermore, tests used to measure the amount of body fat, such as dual-energy X-ray absorptiometry (DEXA) and bioimpedance analysis, are not widely available, which limits the suitability of this criterion; DEXA devices have weight limits, which may prevent adequate patient accommodation, show relatively low concordance in estimating body fat, and there is no standardization across different methods and devices, which may affect diagnostic acuracy [67, 68]. It is important to recognize that the use of these devices is not mandatory for the diagnosis of obesity [64].

The new criteria may offer several advantages, such as allowing the diagnosis of obesity in individuals with excess adiposity, even in individuals with a BMI < 30 kg/m^2^ (measured by other criteria mentioned above), or preventing the misclassification of obesity (e.g., in individuals with water retention or high muscle mass). On the other hand, elevated body fat was included as an independent and sufficient diagnostic criterion for diagnosing obesity, but no threshold was provided [64].

Nevertheless, a recent study evaluated the prevalence of obesity with and without confirmation of excess adiposity in US adults to assess the population-level implications and clinical utility of this confirmatory approach. The prevalence of obesity (assessed by BMI only) was compared with the obesity prevalence when excess adiposity was confirmed by WC measurement or DEXA in a sample of 2,225 US adults aged 20 to 59 years from the 2017–2018 National Health and Nutrition Examination Survey (NHANES), the most recent nationally representative study with direct body fat measurement by DEXA. BMI and anthropometric measurements used thresholds adjusted for ethnicity and sex. Participants with either an elevated BMI and an elevated WC, an elevated body fat percentage, or a BMI of 40 or greater were classified as having confirmed excess adiposity (in accordance with the Commission’s definitions). The study compared the prevalence of obesity defined by BMI alone with the prevalence obtained using the Commission’s confirmatory criteria for excess adiposity. These estimates should be interpreted within the context of the Commission’s operational definition and the nationally representative NHANES sample from which they were derived. It also examined how many individuals meeting BMI criteria for obesity actually had confirmed excess adiposity. The prevalence of obesity was nearly identical when defined by elevated BMI only (39.7%) or when defined by elevated BMI in conjunction with criteria for excess adiposity confirmation (39.1%). Overall, 98.4% of persons with obesity assessed by BMI only had confirmed excess adiposity (97% of men and 99.9% of women), with consistent results across age, sex, and racial and ethnic groups [69].

However, that study evaluated a smaller population and did not incorporate WHR or WHtR. The concurrent use of all three anthropometric criteria alongside BMI likely led to an increase in the estimated prevalence of obesity compared to using a BMI threshold of 30 kg/m^2^ or greater alone. These findings are consistent with previous studies demonstrating a strong population-wide correlation between BMI and excess adiposity in US adults [70, 71]. Although certain populations such as athletes warrant further evaluation, these individuals make up a very small portion of the population [72]. For nearly all US adults with an elevated BMI, confirmation of excess adiposity in routine clinical practice has limited utility, because it requires specialized equipment and incurs out-of-pocket costs. Other anthropometric indices may be convenient and complementary but are unnecessary for patients with a higher BMI [69].

Another cross-sectional study estimated the prevalence of clinical obesity based on the Lancet Commission criteria among US adults across demographic subgroups, using data from the 2017–2020 NHANES cycle, which included 8,037 adults aged 20 years or older. The overall prevalence of clinical obesity was 9.7% (9.8% in males and 9.6% in females). Young adults (20–39 years) had the highest prevalence (11.9%), followed by those aged 40–59 years (8.2%). Mexican females had the highest prevalence (13.9%), followed by non-Hispanic Black females (13.5%) and non-Hispanic White males (10.6%). The prevalence decreased significantly when the new definition incorporating organ dysfunction or limitations in daily activities was applied, compared with BMI alone [73]. The substantial variation in the prevalence of clinical obesity makes it difficult, at this point, to determine how useful this criterion truly is.

Another study aimed to estimate the prevalence of preclinical and clinical obesity using a large and diverse dataset, the NIH All of Us (AoU) Research Program [74]. Using an AoU database of nearly 250,000 adults, anthropometric data—including height, weight, WC, and hip circumference—were measured in person by trained staff using standardized protocols. Following the calculation of BMI, WHR, and WHtR, diagnostic criteria for obesity, preclinical obesity, and clinical obesity were applied. The analysis revealed that 32.0% of the participants did not have obesity, while 68.0% did, with 28.9% classified as preclinical and 39.1% as clinical. This contrasts with the 40.3% prevalence of obesity reported by the WHO based on the 2021–2023 NHANES. These prevalence estimates should not be directly compared with those derived from NHANES because they were generated using different study populations, operational definitions of obesity, and analytical approaches. The substantially higher prevalence observed in the All of Us cohort likely reflects these methodological differences, in addition to differences in participant characteristics.

The presence of preclinical and clinical obesity across BMI categories was, respectively: among individuals with normal BMI, 11.3% and 11%; among those with overweight, 31.8% and 33.5%; among those with class 1 obesity, 31.6% and 55.4%; among those with class 2 obesity, 14.2% and 63.1%; and among those with class 3 obesity, 11% and 70.6%. Therefore, the prevalence of clinical obesity, as expected, increases as BMI increases, while the prevalence of preclinical obesity decreases as the severity of obesity increases [75].

For the diagnosis of clinical obesity, 18 criteria were proposed [64]. These are considered symptoms of obesity, not diseases. The rationale is that a diagnosis should rely on a patient’s signs and symptoms rather than another condition, because diseases have their own specific signs and symptoms. This distinction is sometimes less clear in the Lancet Commission’s definition; for example, while hypertension cannot diagnose obesity, elevated blood pressure can. Likewise, ‘apnea or hypopnea during sleep’ is a symptom that can be used for diagnosis, as opposed to ‘obstructive sleep apnea,’ which is a disease [64].

While a full review of all 18 symptoms is beyond the scope of this article, other controversies exist; for instance, although the link between obesity and type 2 diabetes is well established, it is not a direct diagnostic criterion because it is a disease itself. Similarly, hyperglycemia is only a standalone diagnosis when accompanied by low HDL and high triglyceride levels; and metabolic-associated liver disease is only a diagnostic criterion when fibrosis is already present [64].

The criteria lack clear definitions and rely on arbitrary cutoffs, with the severity of the criteria varying across organ systems. Although an initial proposal does not need to be perfect and can be refined over time, the current proposal contains certain inconsistencies that may compromise diagnostic accuracy for some patients. By omitting mental health considerations, the proposal overlooks a crucial aspect of the well-being and quality of life of people living with obesity, potentially exacerbating issues like depression and anxiety that are often linked to the condition.

### Implications for therapy

The stratification of obesity into clinical and preclinical categories has implications for therapy. When *clinical obesity* is present, the objectives of therapy would be at a minimum, to improve its clinical manifestations and, if feasible, achieve remission, as well as to prevent the development of additional disorders, complications, and end-organ damage through lifestyle, psychological, pharmacological, and surgical interventions. When *preclinical obesity* is diagnosed, the aims would be to prevent progression to clinical obesity and the development of obesity-related diseases through evidence-based health advice and health care [76].

Although a key strength of the classification is its emphasis on avoiding delays in evidence-based treatment for individuals with clinical obesity, this distinction might postpone effective therapy for people with preclinical obesity, particularly because the document states that preclinical obesity is merely a risk factor rather than a disease. Importantly, this contrasts with the widely accepted understanding that, in chronic noncommunicable diseases, prompt early intervention can prevent complications more effectively than waiting until clinical consequences are established—that is, until *clinical obesity* is present—when the objective would be to improve or achieve remission of clinical disorders. Such an approach contrasts with well-established principles of chronic disease care, in which early diagnosis and prevention are considered best practices and the timely implementation of all recommended interventions is strongly endorsed. Of particular concern is the effect on young people, in whom prolonged excess fat accumulation may trigger the earlier onset of metabolic complications.

We understand that the authors state that the proposed diagnostic framework is not a management guideline, and that individuals with preclinical obesity may receive treatment but would probably need to be deprioritized in situations in which treatment is not available to everyone with excess body fat. Certainly, access to obesity treatment remains far from ideal, even for individuals with more evident symptoms and complications of obesity [77]. However, the controversy over whether preclinical obesity is a disease could affect access to care in settings where such care is already available and create the impression that, if even specialists on the field are uncertain whether obesity is a disease, inaction may be justified.

Early and accurate diagnosis of obesity is essential to prevent and treat related comorbidities, halt disease progression, and ensure that patients receive timely and appropriate care without being denied access to treatment [76, 77].

## EASO framework

A group of dissenting members from the Lancet Commission developed an alternative framework under the auspices of the European Association for the Study of Obesity (EASO) [78]. While acknowledging certain strengths of the proposal, the framework adopts a substantially broader definition of obesity. It retains a BMI of 30 kg/m^2^ as the diagnostic threshold, irrespective of complementary assessment methods, and does not incorporate clinical or preclinical staging. In addition, the framework extends the definition to individuals with a lower BMI, classifying individuals as having obesity when their BMI exceeds 25 kg/m^2^ and their WHtR is greater than 0.5, provided that at least one “clinical component” — medical, functional, or psychological — is present. This model places greater emphasis on abnormal fat distribution and underscores the importance of early diagnosis and intervention.

The 2026 update [79] includes results of a randomized phase 3b, open-label, controlled trial comparing the efficacy and safety of semaglutide with tirzepatide in adults with obesity but without type 2 diabetes. This trial showed that 72 weeks of treatment with tirzepatide was superior to treatment with semaglutide in reducing body weight and WC [80]. Additionally, the update presents results from a planned interim analysis conducted at week 72 in the first 800 patients enrolled in an ongoing phase 3, multicenter, randomized, double-blind, placebo-controlled trial. This study assigned over 1,000 patients with biopsy-defined metabolic dysfunction-associated steatohepatitis (MASH) and stage 2 or 3 fibrosis to receive either once-weekly subcutaneous semaglutide (2.4 mg) or a placebo for 240 weeks, demonstrating that semaglutide improved liver histology [81]. Finally, results from a smaller phase 2 trial involving 190 participants with MASH and moderate or severe fibrosis showed that 52 weeks of treatment with tirzepatide was more effective than placebo in achieving MASH resolution without worsening fibrosis [82].

Table 4 compares the traditional anthropometric definitions with those proposed by the Lancet Commission, EASO and Edmonton Obesity Staging System.

**Table 4:** Comparison of traditional anthropometric definitions with the Lancet Commission and edmonton obesity staging system

Classification	Diagnosis of obesity	Clinical staging	
WHO	BMI ≥ 30 kg/m^2^	No	
EASO	BMI ≥ 30 kg/m^2^ or ≥ 25 kg/m^2^ + waist-to-height ratio > 0.5 + a “clinical” component (medical, functional, or psychological)	No	
Lancet commission	BMI ≥ 30 kg/m^2^ or ≥ 25 kg/m^2^ + one anthropometric criterion* or two anthropometric criteria* or direct measurement of body fat or BMI ≥ 40 kg/m^2^	Preclinical	Clinical
EOSS	Not intended for diagnosis	0, 1, 2**	2**, 3, 4

^*^Waist circumference, waist-hip ratio or waist-to-height ratio; ** Depending on the degree of organ and tissue involvement caused by chronic obesity-related disease and the degree of activity limitation; BMI: body mass index; EASO: European Association for the Study of Obesity; EOSS: Edmonton Obesity Staging System; WHO: World Health Organization

Nevertheless, a principal criticism of this framework is that it markedly increases the estimated global prevalence of obesity, which may inadvertently discourage action by policymakers and health care stakeholders, who could argue that providing care to all individuals meeting this expanded diagnostic criterion is unrealistic.

## Obesity classification based on weight history

A complex pathophysiological process drives obesity and weight recurrence , whereby the body defends a weight "set point". Attempts to lose weight trigger counterregulatory responses from the hypothalamus and brainstem, that increase hunger and reduce energy expenditure. This physiological adaptation makes weight recurrence likely after interventions, with no evidence that the "set point" can be reset downward [83, 84].

### Clinical benefits of modest weight loss

Obesity treatment rarely leads to a cure; instead, an achievable weight loss of 5–10% significantly reduces health risks. Treatment is challenging because clinicians often encourage additional, difficult-to-achieve weight loss which increases the risk of weight recurrence [85–87].

Even relatively small weight losses are associated with health and quality-of-life benefits. Weight losses of 3–5% provide health benefits, such as improved glucose control and fertility, whereas losses of 5–10% are considered clinically significant and can improve metabolic markers, mood, joint pain, and sexual function. Weight loss of at least 7% is associated with a lower incidence of type 2 diabetes, whereas losses exceeding 10% are associated with improvements in MASH [85–87]. A nonrandomized post hoc analysis of the Look AHEAD (Action for Health in Diabetes) trial [88] found that, among individuals with type 2 diabetes, achieving an early weight loss of 11% at 1 year was associated with a nearly 21% reduction in myocardial infarction, and a 23% decrease in intra-abdominal fat. However, because these findings derive from a post hoc analysis rather than a prespecified randomized comparison, they should be interpreted cautiously and should not be considered sufficient to support guideline recommendations regarding cardiovascular mortality reduction [89]. The DiRECT trial (Diabetes Remission Clinical Trial), a multicenter open-label, cluster-randomized controlled trial, compared a weight management program with standard care and found that substantial weight loss was associated with type 2 diabetes remission, with 57% of participants achieving remission after a 10% weight loss and 86% after a 15% weight loss at 1 year [89]. The remission rate was 36% at 2 years and declined to 13% at 5 years, a rate that remained notable compared with usual care [90]. The Look AHEAD trial reported a 7% remission at year 4 [91]. Achievable weight loss is recommended as part of obesity treatment, with even modest reductions of approximately 5% of body weight leading to clinically significant health improvements, particularly in metabolic markers, such as insulin action. This degree of weight loss is considered an achievable first step in treatment [92].

### New classification based on the maximum weight attained in life

Consequently, in 2022, the Brazilian Society of Endocrinology and Metabolism (SBEM) and ABESO proposed an obesity classification based on the maximum weight attained in life (MWAL) and the percentage of weight loss to guide treatment and clinical management decisions, rather than solely on current BMI [93]. Importantly, this is an adjunctive classification intended to support clinical assessment and, potentially, research. It is not designed to replace existing systems; on the contrary, it was created to be applied alongside them to enhance clinical decision-making.

This new classification, developed by a group of experts from both Brazilian medical societies, categorizes individuals based on their weight trajectory during nonsurgical clinical treatment for obesity (including nonpharmacological and pharmacological treatments) and is intended for adults aged 18–65 years with BMI values ranging from 30 to < 50 kg/m^2^. The classification does not apply to individuals with end-stage diseases, who are experiencing involuntary weight loss, individuals receiving short-term medications that cause weight gain, individuals receiving corticosteroids chronically or intermittently or individuals with endogenous cortisol production due to Cushing syndrome, altered body composition, or advanced age. In contrast to other proposals for the reclassification of obesity that discourage the use of BMI as the sole diagnostic criterion, this classification adopted BMI starting point for simplicity [93].

According to this classification, patients should be asked at their first visit about their MWAL (excluding weight attained during pregnancy). This value should be used for the primary diagnosis according to the WHO classification of obesity (Table 2; class I, 30.0 to  < 35 kg/m^2^; class II, 35.0 to  < 40 kg/m^2^) followed by the terms “unchanged” (if weight loss is  < 5%), “reduced” (if weight loss is 5% to  < 10%), or “controlled” (if weight loss is  ≥ 10%); the percentage of weight loss (by 5% increments) should also be reported. For individuals with a BMI ​​between 40 and  < 50 kg/m^2^, the term “controlled” should be applied if the weight loss achieved is at least 15%, “reduced” if weight loss is 10% to  < 15%, and “unchanged” if  < 10% (Table 5). For ethnic groups with different BMI cutoff values for defining obesity, the classification can respect the specific criteria. This proposed classification incorporates MWAL as important information to be collected as part of the medical history, facilitating the understanding of obesity as a chronic disease and, regardless of the current weight status, acknowledging the biology tendency toward weight recurrence . A limitation of this classification is its dependence on self-reported maximum weight, which is susceptible to recall bias. Furthermore, its applicability outside the adult BMI range of 30–50 kg/m^2^ is limited, and it requires broader validation across diverse populations [93].

**Table 5:** Proposed classification of “reduced” and “controlled” obesity based on maximum body mass index (BMI)

Maximum BMI	Unchanged	Reduced	Controlled
30 to < 40 kg/m^2^	< 5%	5% to < 10%	≥ 10%
40 to < 50 kg/m^2^	< 10%	10% to < 15%	≥ 15%

One limitation of this classification is that it defines “reduced” and “controlled” solely on the basis of weight loss. However, the primary goal of obesity treatment extends beyond weight reduction to encompass improvements in overall long-term health outcomes and quality of life. A more robust definition of “controlled” would encompass not only weight loss targets but also objective metrics of clinical improvement, such as changes in metabolic parameters, validated patient-reported outcome measures, and the amelioration or remission of obesity-related conditions [93].

In this regard, an appropriate strategy would involve integrating this classification with the Lancet Commission criteria. In individuals presenting with one or more of the 18 obesity-related symptoms, the therapeutic objective could be defined as achieving a specified percentage of weight loss accompanied by demonstrable improvement or remission of these symptoms, thereby signifying a transition from clinical to preclinical obesity in some cases.

Similarly, an international panel of experts representing 21 countries proposed 20 outcome measures to be systematically assessed before and throughout obesity treatment. Incorporating selected, modifiable indicators from this framework alongside weight loss targets would provide a more precise definition of “controlled” obesity. Further conceptual and empirical refinement in this domain is warranted [94].

### Perceptions of people with obesity regarding the classification

This classification can help patients and health care professionals discuss more realistic weight loss goals, and can serve as an adjunct to other measurements, classifications, and clinical findings. The perceptions of 500 people with obesity were explored in a cross-sectional online survey, which revealed that 64% had never been asked by a health care professional about their MWAL. After learning about the new classification, the majority considered it useful for changing perceptions of obesity treatment; felt that it would encourage them to seek treatment; believed that it would help them maintain treatment; and considered that achieving "controlled obesity" would make them feel better even if their total weight loss did not meet their expectations. Furthermore, the majority agreed that the classification could help establish realistic goals and reduce bias among health care professionals, and perceived it as beneficial for encouraging long-term treatment adherence and reducing stigma [95].

## Comparative analysis of classification systems

Table 6 compares the core definitions, strengths, weaknesses, and clinical utility of the obesity classification systems proposed by the WHO, the EOSS, the Lancet Commission, EASO, and the weight-history classification.

**Table 6:** Comparison of the fundamental definitions, strengths, weaknesses, and clinical value of obesity classification systems from the WHO, EOSS, Lancet Commission, EASO, and the weight-history classification

Classification system	Core definition	Strengths	Weaknesses	Clinical utility
WHO classification	Defined by WHO as “abnormal or excessive fat accumulation that presents a risk to health”; operationalized through BMI (weight/height^2^, kg/m^2^), a graded classification (Table 2) supplemented by WC, WHR or WHtR as measures of fat distribution; originally developed and explicitly judged by its own authors as appropriate for population studies but inadequate for individual clinical assessment	Simple, reproducible, inexpensive, and easy to use as a screening tool; globally adopted and standardized, enabling comparisons within and between populations; useful for epidemiological-level studies and public health surveillance, identifying groups at increased risk of morbidity and mortality and providing a basis for evaluating interventions	BMI alone does not provide information on body composition, fat distribution, adipose tissue dysfunction (adiposopathy), presence of comorbidities, or individual risk; insufficient as a sole indicator of health; may under- or overestimate adiposity in certain populations (e.g., athletes, older adults with sarcopenic obesity); standard cut-offs derived from Caucasian populations do not equally apply across ethnicities – South Asian, Black, Chinese and Arab populations show increased comorbidity risk at lower BMI thresholds, and abdominal fat distribution for a given BMI also differs by ethnicity; does not capture dietary quality (e.g., ultra-processed vs. Mediterranean dietary patterns intake), which modifies metabolic risk independent of BMI category; reliability in the elderly is limited by age-related changes in body composition and by reverse causation in short-term studies	Helpful for initial screening and risk assessment at the population level; useful as an initial step alongside WC, WHR or WHtR (WHtR has outperformed BMI and WC in predicting cardiometabolic risk in some cohorts); limited in guiding individual-level clinical decision-making when used in isolation
Edmonton Obesity Staging System (EOSS)	Five-stage (0–4) clinical and functional staging system based on metabolic, mechanical, and mental health complications, not on BMI or the simple presence of increased body fat – 0: no apparent risk factors; 1: subclinical risk factors/mild limitations; 2: established obesity-related chronic disease; 3: established end-organ damage; 4: severe, potentially end-stage disabilities; not intended as a diagnostic tool for obesity itself, but as a complement to anthropometric classification	Integrates metabolic, mechanical and mental health dimensions using simple, accessible clinical evaluations (medical history, clinical and functional assessment, and routine examinations); permits stratification of patients by risk and can help prioritize treatment when resources are limited; validated in a nationally representative NHANES cohort, in which survival curves diverged by EOSS stage but not by obesity class (BMI) alone; higher EOSS scores are also associated with increased perioperative complications after bariatric/metabolic surgery	Requires clinical evaluation and judgment, which may introduce subjectivity; less widely adopted than BMI-based classification; static system – many criteria are nonmodifiable, so it does not reliably capture improvement with treatment, limiting its use for assessing long-term outcomes; causal relationship between some criteria and obesity itself can be questioned (e.g., whether diabetic retinopathy, a stage 3 criterion, is directly caused by obesity and reversible with its treatment); stage 4 could not be reliably assigned in the validation cohort owing to missing data	Useful for clinical decision-making and guiding tailored treatment intensity; helps identify individuals at higher risk of obesity-related complications; superior to obesity class (BMI) alone for mortality prediction; informs perioperative risk assessment in bariatric/metabolic surgery candidates
Definition and diagnostic criteria of clinical obesity	Developed by a Commission of approximately 60 experts (The Lancet Diabetes & Endocrinology and King’s Health Partners, chaired by Francesco Rubino), published in 2025 and endorsed by more than 70 medical societies (including TOS, ADA, WOF and ABESO), but opposed by the OMA and EASO; defines obesity as excess adiposity regardless of tissue distribution or function; distinguishes preclinical obesity (excess adiposity without organ dysfunction) from clinical obesity (a chronic, systemic disease in which excess adiposity causes organ/tissue dysfunction or functional limitations); diagnosis of obesity requires one of three alternative criteria – direct body fat measurement (DEXA or bioimpedance analysis), one anthropometric criterion (WC, WHR or WHtR) plus BMI, or two anthropometric criteria without BMI – or a BMI ≥ 40 kg/m^2^ alone; clinical obesity is further defined by 18 proposed diagnostic criteria describing organ dysfunction or functional impairment, conceived as symptoms rather than diseases in themselves	Product of a high-quality, broadly endorsed scientific process; explicitly moves beyond BMI as the sole diagnostic tool; distinguishes preclinical from clinical obesity, extending the obesity diagnosis to people with BMI < 30 kg/m^2^ who have confirmed excess adiposity; mitigates overdiagnosis in individuals with high muscle mass or fluid retention by requiring confirmatory criteria beyond BMI; recommends use of multiple anthropometric measures	Requires more extensive clinical assessment; the three alternative diagnostic pathways can classify the same individual differently depending on which is applied; DEXA and bioimpedance are not widely available, are not mandatory, involve out-of-pocket costs, have weight limits, and show relatively low concordance between methods; the 18 diagnostic criteria lack precise, standardized cut-offs and their severity varies by organ, which may compromise diagnostic accuracy; the proposal omits mental health criteria despite its relevance to quality of life and stigma; classifying preclinical obesity as a risk factor rather than a disease may delay evidence-based treatment, a particular concern in children and adolescents; estimated prevalence varies substantially across studies; more difficult to apply in population-level studies than BMI alone	Provides a more comprehensive, disease-based approach to diagnosing and managing obesity; clinical obesity warrants active intervention aimed at improving or achieving remission of manifestations and preventing further organ damage; preclinical obesity warrants preventive, evidence-based lifestyle strategies to avert progression; can potentially be integrated with the weight-history (MWAL) classification, e.g., defining transition from clinical to preclinical obesity as weight loss accompanied by symptom remission
The EASO framework	Alternative framework developed by dissenting members of the Lancet Commission under EASO; retains BMI ≥ 30 kg/m^2^ as the dagnostic threshold irrespective of complementary assessment methods and, unlike the Lancet Commission, does not incorporate clinical/preclinical staging; extends the obesity definition to BMI 25–29.9 kg/m^2^ plus WHtR > 0.5 plus at least one “clinical component” (medical, functional, or psychological); the 2026 update also incorporates new trial evidence on incretin-based therapies and MASH treatment	Substantially broader definition, recognizing obesity as a chronic, progressive disease defined by pathological fat accumulation per se, independent of the presence of clinical complications, and extending diagnosis to individuals with lower BMI but increased abdominal fat; places strong emphasis on abdominal fat accumulation as a risk factor beyond BMI; underscores the importance of early diagnosis and intervention; 2026 update cites trial data showing tirzepatide superior to semaglutide for reducing body weight and waist circumference at 72 weeks, and semaglutide and tirzepatide efficacy for MASH histological improvement	Requires additional anthropometric measurement (WHtR) beyond BMI for the expanded (BMI 25–29.9) category; unlike EOSS or the Lancet Commission, does not offer a staging system to grade severity or guide treatment intensity; principal criticism is that it markedly increases the estimated global prevalence of obesity, which could discourage policymakers and health systems from committing to providing care to all who meet the expanded criteria	Helps identify individuals at higher cardiometabolic risk even with lower BMI; encourages a more comprehensive, person-centered approach to obesity management focused on long-term health outcomes; supports early diagnosis and intervention
Obesity classification based on weight history	Proposed in 2022 by SBEM and ABESO; based on the maximum weight attained in life (MWAL, excluding pregnancy) and the percentage of weight loss from that maximum, rather than on current BMI alone; conceived as an adjunct to, not a replacement for, existing systems; applies to adults aged 18–65 years old with BMI 30 to < 50 kg/m^2^ undergoing non-surgical treatment; primary diagnosis still uses the WHO BMI classes, qualified as “unchanged” (< 5% loss), “reduced” (5%– < 10%) or “controlled” (≥ 10%) for BMI 30– < 40 kg/m^2^, and “unchanged” (< 10%), “reduced” (10%– < 15%) or “controlled” (≥ 15%) for BMI 40– < 50 kg/m^2^; excludes individuals with involuntary weight loss from end-stage disease, corticosteroid use, or Cushing syndrome	Recognizes that modest, achievable weight loss (e.g., 5–15%) provides significant clinical benefit regardless of final BMI, and values the MWAL as information that captures the biology of weight recurrence and the “set point,” framing obesity as a chronic, relapsing disease; encourages focus on weight maintenance and prevention of recurrence rather than pursuit of maximal loss; may reduce weight stigma; in a survey of 500 people with obesity, most found the classification useful for reframing treatment goals and encouraging adherence, and 64% had never previously been asked about their MWAL by a health professional	Defines “reduced” and “controlled” status solely by percentage weight loss, without incorporating metabolic parameters, patient-reported outcomes, or symptom remission; depends on self-reported MWAL, which is susceptible to recall bias; applicability is restricted to adults with BMI 30 to < 50 kg/m^2^ and excludes several clinical scenarios (e.g., corticosteroid-induced or Cushing syndrome-related weight gain, older age, altered body composition); still undergoing validation; less widely recognized and adopted than BMI-based classification; requires broader validation across diverse populations	Can be used as an adjunct to other classification systems to provide a more complete picture of an individual’s weight history and associated risk, supporting realistic goal-setting and long-term adherence; proposed integration with Lancet Commission criteria could define “controlled” obesity as a transition from clinical to preclinical status; incorporating outcome measures from a 21-country expert panel could further refine the definition of “controlled” obesity

ABESO: Brazilian Association for the Study of Obesity and Metabolic Syndrome; ADA: American Diabetes Association; BMI: body mass index; DEXA: dual-energy x-ray absorptiometry; EASO: European Association for the Study of Obesity; MASH: metabolic-associated steatohepatitis; MWAL: maximum weight attained in life; NHANES: National Health and Nutrition Examination Survey; OMA: Obesity Medical Association; SBEM: Brazilian Society of Endocrinology and Metabolism; TOS: The Obesity Society; WC: waist circumference; WHR: waist-hip ratio; WHtR: waist-to-height ratio; WOF: World Obesity Federation; WHO: World Health Organization

While these frameworks attempt to capture the clinical heterogeneity of obesity, they remain heavily reliant on clinical staging and epidemiological associations rather than underlying disease biology. A comprehensive understanding of obesity as a chronic disease ultimately requires definitions that transcend simple anthropometric phenotypes and address its complex pathophysiology and molecular drivers, including biomarkers of adipose tissue dysfunction (e.g., ectopic fat deposition, macrophage infiltration, altered adipokine secretion, and pro-inflammatory signaling) rather than just the history of weight gain.

## Conclusion

BMI was adopted by the WHO almost three decades ago and is a valuable measure for assessing obesity at the population level. It can still be useful at the individual patient level, especially when used in conjunction with other anthropometric measurements and with sex-, age-, and ethnicity-specific cutoff values. In turn, the EOSS is a simple clinical and functional staging system that distinguishes the severity and consequences of obesity and can guide the intensity of appropriate interventions more accurately than BMI in children, adolescents and adults. The definition of obesity and the diagnostic criteria for preclinical and clinical obesity proposed by a committee of experts generated some disagreement but received the endorsement of dozens of medical societies. The main concerns were potential difficulties in achieving an accurate diagnosis because of the lack of standardized screening tools, as well as possible delays in treatment for people with preclinical obesity. Both diagnostic inaccuracy and delayed intervention may be particularly harmful to children and adolescents living with obesity. Finally, the weight-history classification complements the aforementioned frameworks. It incorporates information on MWAL, supports the establishment of realistic goals based on maximum BMI, recognizes obesity as a chronic disease, helps reduce bias and stigma, and encourages treatment adherence.

## Data Availability

No datasets were generated or analysed during the current study.

## Abbreviations

ABESO: Brazilian association for the study of obesity
AHEAD: Action for health in diabetes
AoU: All of Us research program
BMI: Body mass index
CPRD: Clinical practice research datalink
DEXA: Dual-energy X-ray absorptiometry
DiRECT: Diabetes remission clinical trial
EASO: European association for the study of obesity
EOSS: Edmonton obesity staging system
KDOQI: Kidney disease outcomes quality initiative
MASH: Metabolic-associated Steatohepatitis
MWAL: Maximum weight attained in life
NHANES: National health nutrition examination survey
NIH-AARP: National institute of health-american association of retired persons
NYHA: New York heart association
SBEM: Brazilian society of endocrinology & metabolism
TNM: Tumor, node, metastasis
US: United States
WC: Waist circumference
WHO: World Health Organization
WHR: Waist-hip ratio
WHtR: Waist-to-height ratio
